# A heat-shocked melanoma cell lysate vaccine enhances tumor infiltration by prototypic effector T cells inhibiting tumor growth

**DOI:** 10.1136/jitc-2020-000999

**Published:** 2020-07-20

**Authors:** María Alejandra Gleisner, Cristián Pereda, Andrés Tittarelli, Mariela Navarrete, Camila Fuentes, Ignacio Ávalos, Fabian Tempio, Juan Pablo Araya, María Inés Becker, Fermín Eduardo González, Mercedes Natalia López, Flavio Salazar-Onfray

**Affiliations:** 1 Disciplinary Program of Immunology, Institute of Biomedical Sciences, Faculty of Medicine, Universidad de Chile, Santiago, Chile; 2 Millennium Institute on Immunology and Immunotherapy, Faculty of Medicine, Universidad de Chile, Santiago, Chile; 3 Programa Institucional de Fomento a la Investigación, Desarrollo e Innovación, Universidad Tecnológica Metropolitana, Santiago, Chile; 4 Fundación Ciencia y Tecnología para el Desarrollo (FUCITED), Avenida Eduardo Castillo Velasco 2902, Santiago, Chile; 5 Biosonda Corporation, Avenida Eduardo Castillo Velasco 2902, Santiago, Chile; 6 Laboratory of Experimental Immunology & Cancer, Faculty of Dentistry, Universidad de Chile, Santiago, Chile

**Keywords:** immunotherapy, active, melanoma, immunogenicity, vaccine, alarmins, therapies, investigational

## Abstract

**Background:**

Immune checkpoint blocker (ICB) therapy has shown survival benefits for some patients with cancer. Nevertheless, many individuals remain refractory or acquire resistance to treatment, motivating the exploration of complementary immunotherapies. Accordingly, cancer vaccines offer an attractive alternative. Optimal delivery of multiple tumor-associated antigens combined with potent adjuvants seems to be crucial for vaccine effectiveness.

**Methods:**

Here, a prototype for a generic melanoma vaccine, named TRIMELVax, was tested using B16F10 mouse melanoma model. This vaccine is made of heat shock-treated tumor cell lysates combined with the *Concholepas concholepas* hemocyanin as adjuvant.

**Results:**

While B16F10 lysate provides appropriate melanoma-associated antigens, both a generic human melanoma cell lysate and hemocyanin adjuvant contributes with danger signals promoting conventional dendritic type 1 cells (cDC1), activation, phagocytosis and effective antigen cross-presentation. TRIMELVax inhibited tumor growth and increased mice survival, inducing cellular and humoral immune responses. Furthermore, this vaccine generated an increased frequency of intratumor cDC1s but not conventional type 2 dendritic cells (cDC2s). Augmented infiltration of CD3^+^, CD4^+^ and CD8^+^ T cells was also observed, compared with anti-programmed cell death protein 1 (PD-1) monotherapy, while TRIMELVax/anti-PD-1 combination generated higher tumor infiltration of CD4^+^ T cells. Moreover, TRIMELVax promoted an augmented proportion of PD-1^lo^ CD8^+^ T cells in tumors, a phenotype associated with prototypic effector cells required for tumor growth control, preventing dysfunctional T-cell accumulation.

**Conclusions:**

The therapeutic vaccine TRIMELVax efficiently controls the weakly immunogenic and aggressive B16F10 melanoma tumor growth, prolonging tumor-bearing mice survival even in the absence of ICB. The strong immunogenicity shown by TRIMELVax encourages clinical studies in patients with melanoma.

## Background

Immunotherapies based on immune checkpoint blockers (ICBs), targeting inhibitory immune pathways such as cytotoxic T lymphocyte-associated protein 4 or programmed cell death protein 1 (PD-1), have shown significant success in promoting tumor regression and prolonging survival in patients with cancer, particularly in melanoma and other solid tumors.^1 2^ However, many patients do not respond or develop resistance to these interventions, bringing the scientific communities to focus their efforts in combinatorial therapies.^3^ A major factor involved in initial resistance to ICB is lack or weak T-cell tumor infiltration, characterizing the so-called “cold tumors.” In fact, high lymphocyte infiltration and interferon-γ status related to a T-cell inflamed phenotype (hot tumors) constitute key factors for effective anti-PD-1/PD-L1 (programmed cell death ligand 1) therapies.^4 5^ For this reason, immunological treatments that induce adaptive cellular responses in cold tumor patients may be a desirable goal. In this context, active immunotherapies become once again an attractive alternative and/or complement for cancer treatment.^6^

Different strategies for cancer vaccines have been proposed in the past, including those based on recombinant antigenic polypeptides or proteins, modified whole-tumor cells, dendritic cells (DCs) loaded with tumor-associated antigens (TAAs) or DNA vaccines, with dissimilar effects.^6^ Interestingly, some DC vaccines have demonstrated promising results, such as the Food and Drug Administration-approved personalized immunotherapy against advanced prostate cancer Sipuleucel-T (Provenge).^7^ Meanwhile, our group has conducted a series of clinical trials using a DC-based vaccine named tumor antigen-presenting cells (TAPCells),^8 9^ consisting in monocyte-derived antigen-presenting cells (APCs) loaded with an allogeneic heat shock (HS)-conditioned melanoma cell lysate named TRIMEL. These clinical studies showed that ~60% of vaccinated patients developed a delayed-type hypersensitivity (DTH) response against TRIMEL antigens, correlating with a threefold improved overall survival (median: 33 months) compared with DTH-negative patients (median: 11 months).^8 9^ TRIMEL was generated from a mixture of three human melanoma cell lines expressing several known melanoma-associated antigens (MAAs) such as MART-1/MelanA, gp100, tyrosinase, NY-ESO-1, MAGE, GAGE, MC1R, among others.^9^ Interestingly, HS treatments of tumor cells previous to lysis cause plasma membrane translocation of calreticulin and the release of high-mobility group box-1 (HMGB1) and ATP, acting as danger-associated molecular patterns (DAMPs) that induce optimal DC maturation and tumor antigen cross-presentation.^9 10^

Despite encouraging clinical outcomes, biological diversity among patient cells used for DC vaccines^11 12^ and technological challenges related to personalized medicine^13^ have hindered the massive translation of TAPCell therapy into the clinic. For that reason, we propose to bypass these limitations by using a generic therapeutic melanoma vaccine prototype, based on the immunogenic TRIMEL lysate combined with an appropriate adjuvant, which do not require individual adapted manufacturing.

At this respect, previous experimental models using B16F10 melanoma whole cells or derived cell lysates as vaccines generated protective antitumor immune responses only when melanoma cells were genetically modified for expressing strong antigens (ie, ovalbumin), releasing different cytokines (ie, granulocyte-macrophage colony-stimulating factor (GM-CSF)) and/or when combined with ICB.^14–16^ An outstanding example is the GM-CSF gene-transduced tumor vaccine referred to as GVAX, which demonstrated antitumor activity in preclinical cancer models, including the low antigenic B16 melanoma.^17^ However, GVAX and other whole-tumor cell cancer vaccines have shown low objective response rates and very limited improvements of patient survival, suggesting suboptimal immunogenic potential of these approaches, particularly in melanoma.^17–19^ This apparently weak immunogenicity can be due to several reasons, including the absence of appropriate immunological danger signals during immunizations, the suboptimal activation of DCs and/or an inefficient delivery of relevant TAA to resident DC populations responsible for ideal cross-priming of protective CD8^+^ T cells.^20–22^

In this report, we tested a vaccine named TRIMELVax consisting in HS-conditioned TRIMEL plus B16F10 cell lysates combined with *Concholepas concholepas* hemocyanin (CCH) as an adjuvant. The earliest discovered hemocyanin commonly known as keyhole limpet hemocyanin (KLH) is purified from the giant keyhole limpet gastropod *Megathura crenulata* and has been successfully used as an adjuvant in many clinical protocols.^23^ Meanwhile, CCH is a high molecular weight glycoprotein related to oxygen transport in mollusks. It has been described that the highly complex CCH molecule shows a structural stability that contribute to their potent immunostimulatory effects,^24^ strengthening innate and adaptive immunity in mammals and making it useful in cancer immunotherapy.^25 26^ Although both hemocyanins show comparable immunogenic characteristics,^27^ CCH is made up of intermixed subunits organized as heterododecamers that do not require divalent ions, given greater comparative stability. Unlikely, KLH is formed by homododecamers, whose stability depends on Ca^+2^ and Mg^+2^ for maintenance.^25^

Our results showed that TRIMELVax immunizations activate effective cell-mediated and humoral immune responses against B16F10 tumors, inhibiting tumor growth and prolonging mice survival, even in the absence of combined therapy with anti-PD-1 antibodies.

## Methods

### Mice

Wild-type C57BL6, pMEL-1 (JAX stock no: 005023) and Nonobese diabetic/severe combined immunodeficiency (NOD-SCID) (JAX stock no: 005557) mice were maintained at animal facility of the Universidad de Chile, Faculty of Medicine. pMEL-1 mice have C57BL6 background and NOD-SCID mice were on NOD/ShiLtSz background. For all experiments, mice between 6 and 12 weeks of age were bred in specific pathogen-free conditions.

### Cell culture media and reagents

Tumor cell lines were maintained in RPMI-1640 (Corning) supplemented with 1% penicillin/streptomycin (Corning) and 10% heat-inactivated fetal bovine serum (FBS) (Corning). Bone marrow-derived DC (BM-DC) were cultured in RPMI-1640-GlutaMAX (Gibco) supplemented with 1% penicillin/streptomycin, 55 µM 2-mercaptoethanol (Gibco) and 10% FBS. Fluorescence-activated cell sorting (FACS) buffer was phosphate-buffered saline (PBS) (Corning), supplemented with 2% FBS and 2 mM EDTA (Ambion). CCH *Inmunocyanin* was provided by Biosonda. The gp100_25-33_ peptide (KVPRNQDWL) was purchased from Genetel Laboratories. Dr Fabiola Osorio (Universidad de Chile) kindly provided FMS-like tyrosine kinasa 3-ligand (FLT3-L). Lipopolysaccharide (LPS), phorbol myristate acetate and ionomycin were purchased from Sigma-Aldrich. Brefeldin-A was from eBioscience.

### Cell lines and cell lysates

Mel1, Mel2 and Mel3 are human melanoma cell lines isolated from metastatic lymph nodes (LNs) of three patients.^9^ B16F10 (ATCCCRL-6475) and MC38 (ATCCCRL-2639) cells were kindly provided by Dr Álvaro Lladser (Fundación Ciencia & Vida, Chile). FLT3L-expressing B16F10 cells (B16-FLT3L) were kindly provided by Dr María Rosa Bono (Universidad de Chile).

Cell lysates were made as previously described.^9^ TRIMEL is derived from a mix of equal amounts of Mel1, Mel2 and Mel3 cells, which were taken to a final concentration of 8×10^6^ cells/mL, HS treated at 42°C for 1 hour plus 2 hours at 37°C and then lyzed through three cycles of freeze/thaw in liquid nitrogen. The HS-conditioned lysate from B16F10 and MC38 cells were prepared using same approach (8×10^6^ cells/mL). Tumor cells not HS treated were incubated for 3 hours at 37°C before being lyzed.

### Tumor challenge and vaccinations

For therapeutic assays, C57BL6 mice were subcutaneously inoculated with 2.5×10^4^ B16F10 or 1×10^5^ MC38 cells in lower right flanks. Mice were then immunized subcutaneously on lower left flanks on days 1, 6 and 12 post-tumor challenge with corresponding treatments: (1) lysates of B16F10 cells with or without CCH (25 µg/µL, total 200 µg CCH/doses), (2) lysates of HS-conditioned B16F10 cells±CCH, (3) 1:1 mixture of B16F10 cell lysate (preconditioned or not with HS) with TRIMEL±CCH, (4) 1:1 mixture of HS-conditioned MC38 cell lysate with TRIMEL±CCH, (5) CCH or (6) PBS. Each tumor cell lysate doses contained approximately 1.4×10^6^ cells in 172 µL. The mixture of HS-conditioned B16F10 cell lysate plus TRIMEL and CCH corresponds to TRIMELVax. For combination therapy assays, 200 µg/dose of anti-PD-1 (CD279) monoclonal antibody (RMP1-14 (BioXCell)) was intraperitoneally administered on days 4, 7 and 11 post-tumor challenges. For survival assays, mice were sacrificed when tumor growth exceeded 1500 mm^3^. On day 14 after challenge, mice were sacrificed, and tumors, tumor draining LNs (TdLNs) and serum were analyzed.

For prophylactic assays, C57BL6 or NOD-SCID mice were vaccinated subcutaneously at lower left flanks with TRIMELVax or PBS on days −19, −9 and −2 before tumor challenge. On day 0, 1.5×10^5^ B16F10 cells were subcutaneously inoculated at right flanks and tumor growth was measured every 2–3 days.

### Flow cytometry and cell sorting

Antibodies for flow cytometry (BD Pharmigen, eBioscience, BioLegend or Miltenyi Biotec), and viability dye LIVE/DEAD (Thermo Fisher Scientific) were used for cell phenotyping. Depending on the experiment, cells were stained with following antibodies in presence of CD16/31 (Fc-Block): CD11b (M170), I-A/I-E (M5/114.15.2), XCR1 (ZET), CD8a (53.6.7), CD3 (17A2), B220 (RA3-6B2), CD103 (2E7), CD11c (N418), CD69 (IM7), CD24 (M1/49), CD172a (P84), CD45.1 (A20), NK1.1 (PK136), CD4 (GK1.5), CD64 (X54-5/7.1), F4/80 (BM8), CD86 (GL-1), CD24 (M1/69), CD44 (IM7) and PD-1 (29F.1A12). Acquisition and analysis of cell suspensions were performed on FACSVerse and LSR Fortessa X-20 (BD Biosciences) and subsequent analysis was made with FlowJo10 software (FlowJo). Cell sorting was performed on FACS Aria III (BD Biosciences).

### In vitro differentiation of BM-DCs

BM-DCs differentiated in vitro using FLT3-L (FL-DCs) from BM precursor cells of C57BL6 mice femurs and tibias were cultured in BM-DC culture media in presence of 150 ng/mL of human recombinant FLT3-L (PeproTech) for 7–8 days before use.

### Phagocytosis assay

Prior to HS treatment and lysis, mixtures of Mel1/Mel2/Mel3 and/or B16F10 cells were stained with 2 µM PKH26 (Sigma-Aldrich) following manufacturer’s instructions. HS-conditioned PKH26-prelabeled melanoma cell lysates were generated as previously described. 1×10^5^ FL-DCs were cocultured with the stained melanoma cell lysates in 1:1 tumor cell:DC ratio for 0 or 3 hours at 37°C or 4°C. After coculture, lysate capture was determined gating in each FL-DC population using flow cytometry. The phagocytic indexes were calculated as: (%phagocytosis at 37 °C/%phagocytosis at 4°C)×100.

### Activation of FL-DC in vitro

1×10^5^ FL-DCs were stimulated in 1:1 ratio (taking into account the pre-lysis melanoma cell numbers) with TRIMELVax, TRIMEL, HS-conditioned lysates from B16F10 cells, TRIMEL plus HS-conditioned lysate of B16F10 cells, CCH (200 µg), LPS (100 ng/mL) or kept unstimulated for 24 hours. CD86 expression level was analyzed by flow cytometry.

### In vitro and ex vivo antigen cross-presentation assays

For proliferation assays, CD8^+^ T cells were isolated from pMEL-1 splenocytes by negative selection, using a lineage depletion cocktail of biotinylated antibodies and antibiotin microbeads (Miltenyi Biotec) and labeled with 5 µM CellTrace Violet (CTV) (Thermo Fisher Scientific). For in vitro assays, FL-DCs were treated 1:1 with TRIMEL plus HS-conditioned B16F10 cell lysate and CCH (200 µg) for 5 hours at 37°C. For ex vivo assays, inguinal, axillary and brachial LNs were collected from B16-FLT3L tumor-bearing mice. Plasmacytoid DC (pDC), conventional type 1 DC (cDC1) and conventional type 2 DC (cDC2) were isolated by cell sorting. For T-cell activation assays, FL-DCs were collected, washed with FACS buffer and fixed with 1% paraformaldehyde for 10 min. Cells were washed with 0.2M glycine and culture media prior coculture with CD8^+^ T cells. 1×10^5^ fixed DCs were cultured with 1×10^5^ T cells at 37°C for 16 hours to evaluate CD69 or CD25 expression. For proliferation assays, FL-DCs or LN-DCs were collected and immediately cocultured in a 1:1 ratio with CTV-labeled T cells. After 3 days, proliferation was measured by flow cytometry.

### In vivo antigen cross-presentation assays

On day 0, 2×10^6^ CTV-stained pMEL-1 CD8^+^ T cells were intravenously transferred into C57BL6 mice. On days 1, 4 and 7, mice were subcutaneously injected with 20 µg of hgp100 peptide plus CCH (200 µg), TRIMELVax, B16Vax, CCH or PBS. On day 10, inguinal, brachial and axillary LNs were processed and transferred T-cell proliferation was measured by flow cytometry.

### Immunohistochemistry

Tumors were collected and fixed in 4% formalin. Three-micrometer-thick sections from paraffin-embedded tissues were mounted on glass slides, rehydrated and antigen retrieval was performed at 100°C (TRIS-Borate-EDTA (TBE), pH 8). Primary antibodies were used according to manufacturer’s instructions (Abcam, dilutions 1:200): anti-CD3 (SP7), anti-CD4 (EPR19514), anti-CD8 (ab203035). Slides were incubated with primary antibodies in moist chambers overnight at 4°C. Slides were washed with PBS before incubation with label-secondary goat antirabbit IgG for 1 hour at 4°C, washed three times, incubated with DAB substrate KIT (Abcam) solution for 10 min and washed with PBS. Background staining was performed with Mayer’s hematoxylin. Sections were dehydrated through ascending alcohols-to-xylene and mounted.

### Humoral response assay

Indirect ELISA was used to detect antibodies against CCH in sera of treated mice.^28^ Antibodies against melanoma cells or peripheral blood leucocytes (PBLs) were analyzed by flow cytometry. In brief, 1×10^5^ B16F10 or Mel1, Mel2 and Mel3 cell mixtures were incubated with 1:20 dilutions of de-complemented sera. Cells were then incubated with FITC-conjugated anti-mouse IgG (Invitrogen). The binding of serum IgG to target cells was evaluated by flow cytometry.

### Preparation of tumor and LN suspensions

Tumors were dissected, mechanically disaggregated and digested at 37°C for 30 min in Hanks’ balanced salt solution (5% FBS) containing collagenase-D (1 mg/mL) and DNase I (25 µg/mL) (Roche). The cell suspensions were filtered with 70 µm cell strainers (BD Falcon). Leucocytes were suspended in 40% Percoll (GE Healthcare) and gently layered over 70% Percoll. The gradient was centrifuged at 750 g for 20 min at room temperature. Mononuclear cells were collected, washed and resuspended in complemented RPMI-1640. TdLNs were mechanically disaggregated and cells incubated for 45 min at 37°C in a solution containing 100 µg/mL collagenase-D and 50 µg/mL DNaseI dissolved in 2% FBS-PBS. Single cell suspensions were washed in RPMI-1640 and depleted of erythrocytes using RBC lysis buffer (BioLegend) for 5 min at 4°C.

### Statistical analysis

Differences between groups were analyzed by paired, two-tailed Student’s t-tests or Mann-Whitney test. Animal survival data were analyzed with the Kaplan-Meier method. Results with a p-value lower than 0.05 were considered significant. Mean values, SEM and statistics were calculated using GraphPad Prism Software.

## Results

### HS-conditioned melanoma cell lysates are phagocyted by murine cDCs inducing cross-presentation of MAA in vitro

**Figure 1:**
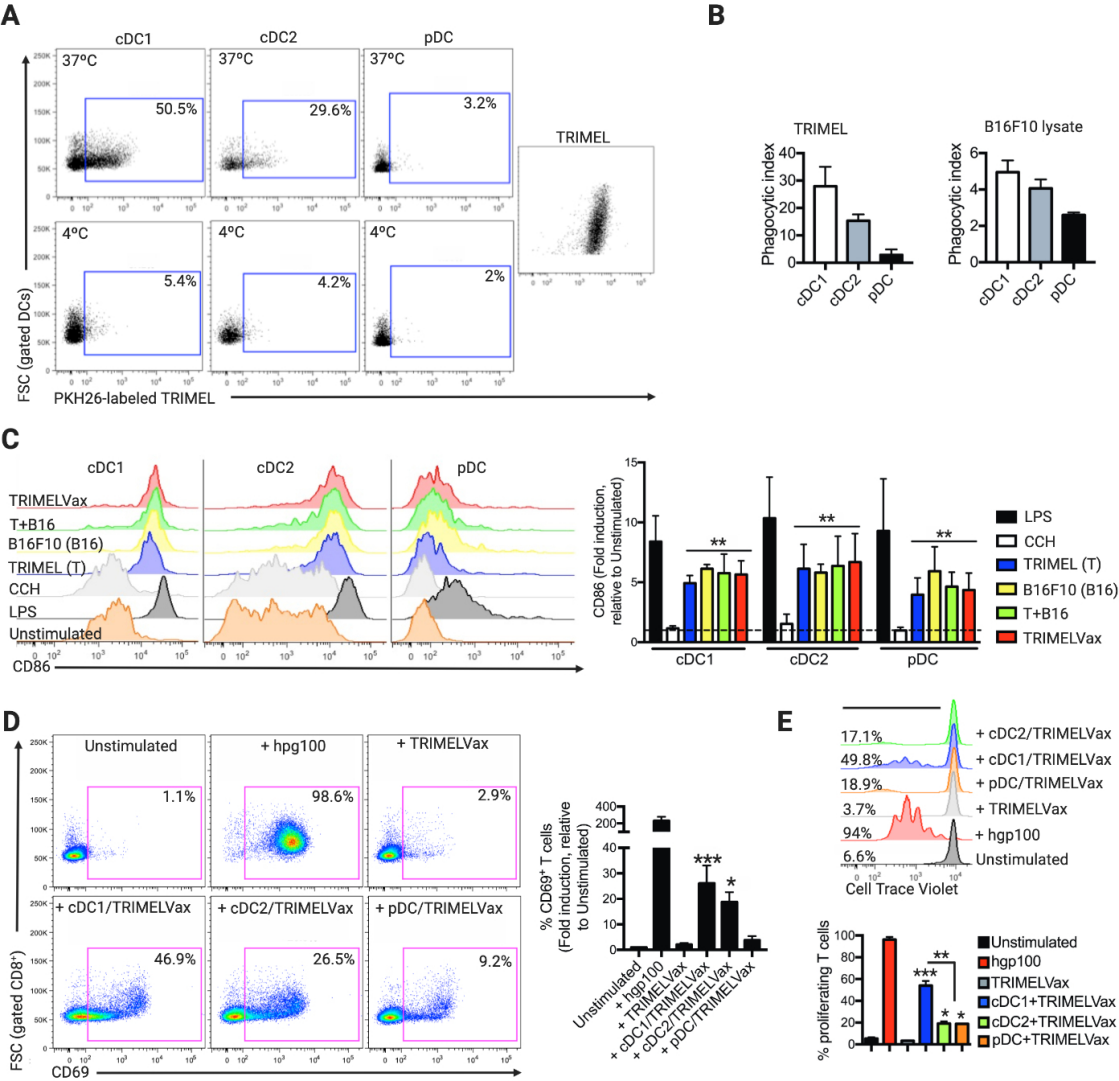
Heatshock (HS)-conditioned melanoma cell lysates are phagocyted by conventional type 1 dendritic cells (cDC1) inducing cross-presentation of melanoma-associated antigen (MAA) in vitro. (A) Bone marrow dendritic cells differentiated with FLT3-L (FL-DCs) were incubated with PKH26-pre-stained TRIMEL (A, B) or B16F10 HS-conditioned cell lysates. Foward scatter (FSC) relative to CD69 expression was analyzed by flow cytometry (B). The percentages of cDC1, conventional type 2 dendritic cell (cDC2) and plasmacytoid dendritic cell (pDC) FL-DCs acquiring PKH26-labeled TRIMEL are shown in representative dot plots. (B) The bar graphs show the average phagocytic index for three independent experiments using TRIMEL (left) or B16F10 HS-conditioned cell lysate (right). (C) FL-DCs were stimulated with lipopolysaccharide (LPS), *Concholepas concholepas* hemocyanin (CCH), TRIMEL (T), B16F10 HS-conditioned cell lysate (B16), a 1:1 mixture of TRIMEL:B16F10 HS-conditioned cell lysate (T+B16), TRIMELVax or kept unstimulated. The expression level of CD86 was analyzed by flow cytometry on FL-DCs. The bar graphs (right) show CD86 expression as fold-change relative to unstimulated FL-DCs. (D) pMEL-1 CD8^+^ T cells were incubated with the peptide gp100_25-33_, TRIMELVax, with sorted and fixed TRIMELVax-loaded FL-DCs or kept unstimulated. CD69 expression was evaluated by flow cytometry. The bar graph (right) shows CD69 expression as fold-change relative to unstimulated T cells. (E) CellTrace Violet (CTV)-preloaded pMEL-1 CD8^+^ T cells were incubated with gp100_25-33_ peptide, TRIMELVax, sorted TRIMELVax-loaded FL-DCs or kept unstimulated. Representative histograms (upper) and graph bars (lower) show the percentage of proliferating CD8^+^ T cells. Statistical analysis was performed with two-way analysis of variance after Bonferroni correction. *p<0.05; **p<0.01; ***p<0.001.

Here, we investigated the effect of TRIMELVax vaccination using the aggressive murine B16F10 melanoma model (online supplementary figure 1). Taking into account the crucial role of DCs in antitumor responses induced by immunizations, first we asked whether mice BM-DCs were capable to sense TRIMEL-originated DAMPs and to phagocyte, process and present MAAs. Our results showed that BM-derived FLT3-L-differentiated DCs (FL-DCs) (online supplementary figure 2) effectively phagocytized both, TRIMEL and B16F10 HS-conditioned cell lysates. In particular, conventional FL-DCs successfully endocyted tumor lysates, while pDC FL-DCs showed lower phagocytic capacity (figure 1A, B). Competitive phagocytosis assays showed that HS-treated B16F10 cell lysates were effectively internalized by cDC1 FL-DCs when incubated in presence of TRIMEL and CCH, suggesting that they may be capable to acquire murine MAA from vaccine components (online supplementary figure 3). Additionally, HS treatment of B16F10 cells also induced ATP and HMGB1 release and calreticulin plasma membrane translocation, similarly to what was observed in HS-treated cell lines composing TRIMEL (online supplementary figure 4).^9 10^ All the FL-DC subtypes increased CD86 expression when pulsed with TRIMELVax or with each HS-conditioned cell lysate, separately or combined, while CCH failed to induce similar activation (figure 1C).

10.1136/jitc-2020-000999.supp1 Supplementary data

Optimal delivery of a wide-ranging pool of TAA coupled with factors promoting antigen cross-presentation seems to be critical for cancer vaccine success. TRIMELVax-stimulated FL-DCs induced cross-presentation of the MAA gp100 as indicated by augmented CD69 cell surface expression on pMEL-1 CD8^+^ T cells, in comparison with both CD8^+^ T cells cultured alone or stimulated with TRIMELVax in absence of DCs, an effect that was more clearly observed when using cDC1 FL-DCs (figure 1D). Furthermore, TRIMELVax-stimulated cDC1 FL-DCs triggered a threefold higher proliferation of CD8^+^ T cells compared with cDC2 and pDC FL-DCs (figure 1E). Altogether, our data indicate that TRIMELVax elicits efficient DC activation for MAA cross-presentation to CD8^+^ T cells, particularly mediated by cDC1 FL-DC.

### TRIMELVax induces humoral and cDC1-mediated CD8^+^ T-cell antimelanoma immune responses in vivo

**Figure 2:**
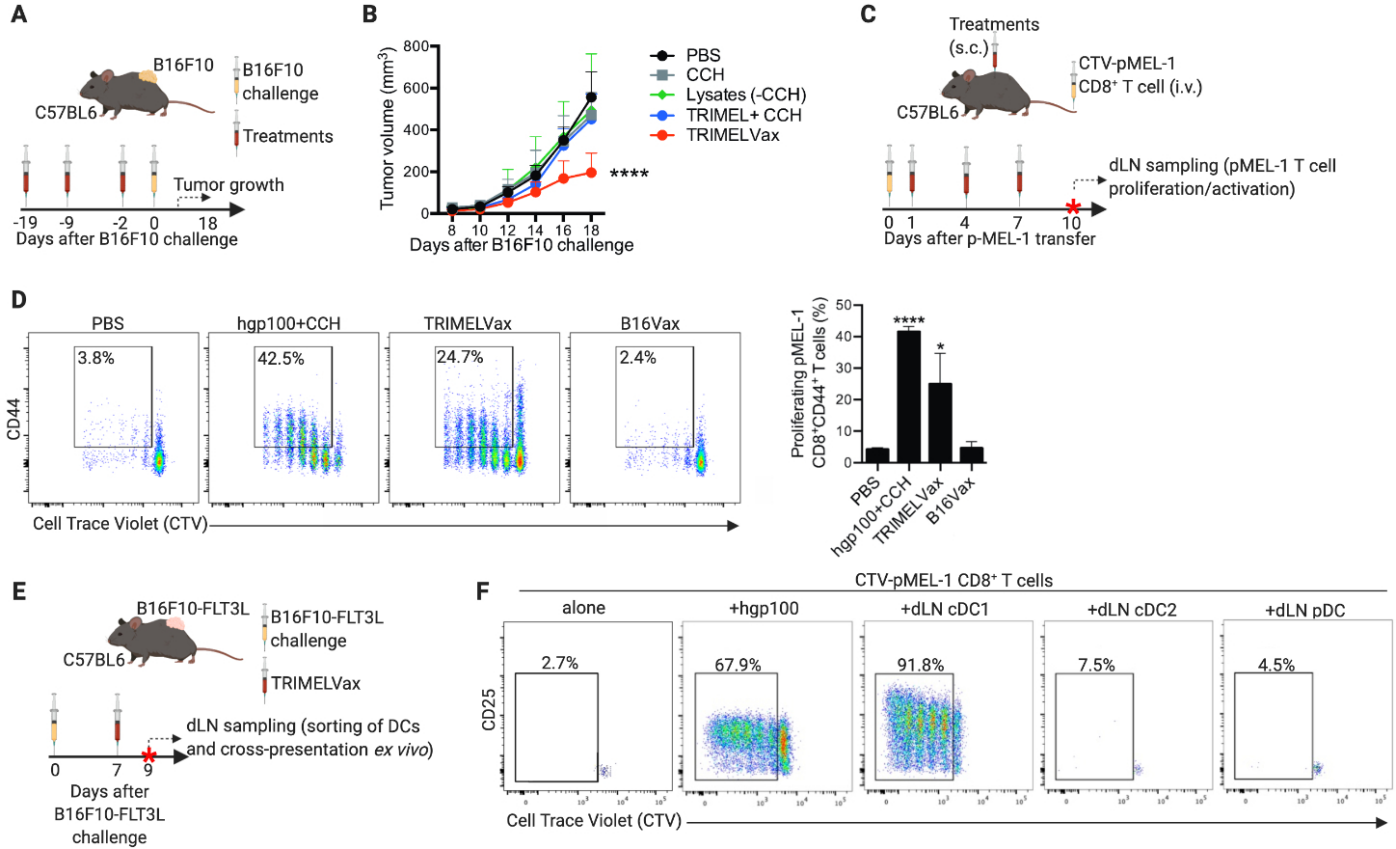
TRIMELVax induces conventional type 1 dendritic cell (cDC1)-mediated CD8^+^ T-cell antimelanoma immune responses in vivo. (A) Schematic representation of prophylactic treatments. (B) Tumor growth curves of mice immunized with different treatments and challenged with B16F10 cells. Each point represents the mean tumor volume±SEM per group (n=7–10). (C) Schematic representation of antigen cross-presentation assay. (D) Proliferation (CellTrace Violet (CTV) dilution) and activation (% of CD44^+^) of CD8^+^ T cells were determined by flow cytometry. (E) Schematic representation of ex vivo antigen cross-presentation assay. (F) CTV-stained pMEL-1 CD8^+^ T cells were cocultured with draining lymph node dendritic cells (dLN-DCs), stimulated with hgp100 peptide or kept unstimulated. The proliferation and activation of the pMEL-1 CD8^+^ T cells was determined after 3 days of coculture. The percentage of proliferating T cells are shown in each representative density plot. Statistical analysis was performed with two-way analysis of variance after Bonferroni correction. *p<0.05; ****p<0.0001. CCH, *Concholepas concholepas* hemocyanin; cDC2, conventional type 2 dendritic cell; PBS, phosphate-buffered saline; pDC, plasmacytoid dendritic cell; s.c., subcutaneously.

Then, we assessed whether immune responses elicited by TRIMELVax in vitro translate in protective antitumor effects in vivo. Mice were immunized with TRIMELVax or control treatments (figure 2A). Two days after the last injection, mice were challenged with B16F10 cells and tumor growth was monitored. In this prophylactic setting, the tumor growth was significantly inhibited only in TRIMELVax-treated mice (figure 2B), indicating that the complete vaccine, but not lysates nor adjuvant alone, induces immune responses able to protect mice against tumor challenge. Moreover, no signals of autoimmunity damage in organs of vaccinated animals were observed (data not shown).

We next investigated whether TRIMELVax-mediated tumor control may be related to eliciting of adaptive antimelanoma immune responses in vivo. To test whether MAA present in TRIMELVax can be efficiently cross-presented to CD8^+^ T cells in vivo, CTV-preloaded pMEL-1 CD8^+^ T cells were transferred intravenously into C57BL6 mice. Mice were treated with TRIMELVax, HS-conditioned B16F10 cell lysate+CCH (B16Vax) or PBS. As a positive control, gp100_25-33_ peptide+CCH were injected 7 days after T-cell transfer. Three days after treatment, dLNs were sampled and proliferation and activation of pMEL-1 CD8^+^ T cells were determined (figure 2C). Our results indicated that TRIMELVax, but not B16Vax, was able to induce potent pMEL-1 CD8^+^ T-cell proliferation and activation (figure 2D). These results strongly suggest that TRIMELVax induces cross-presentation of MAA in vivo promoting a cellular-mediated immune response.

cDC1 cells are the main DC subtype responsible for TAA cross-presentation and antitumor CD8^+^ T-cell activation.^29^ To test whether TRIMELVax was able to provide MAAs to cDC1 in vivo, we performed an ex vivo antigen cross-presentation assay using sorted cDC1, cDC2 and pDCs from TRIMELVax-vaccinated and control B16-FLT3L tumor-bearing animals (figure 2E and online supplementary figure 5). As predicted, only cDC1 cells isolated from dLNs of TRIMELVax-treated but not those isolated from control mice activated pMEL-1 CD8^+^ T cells (figure 2F and online supplementary figure 5).

**Figure 3:**
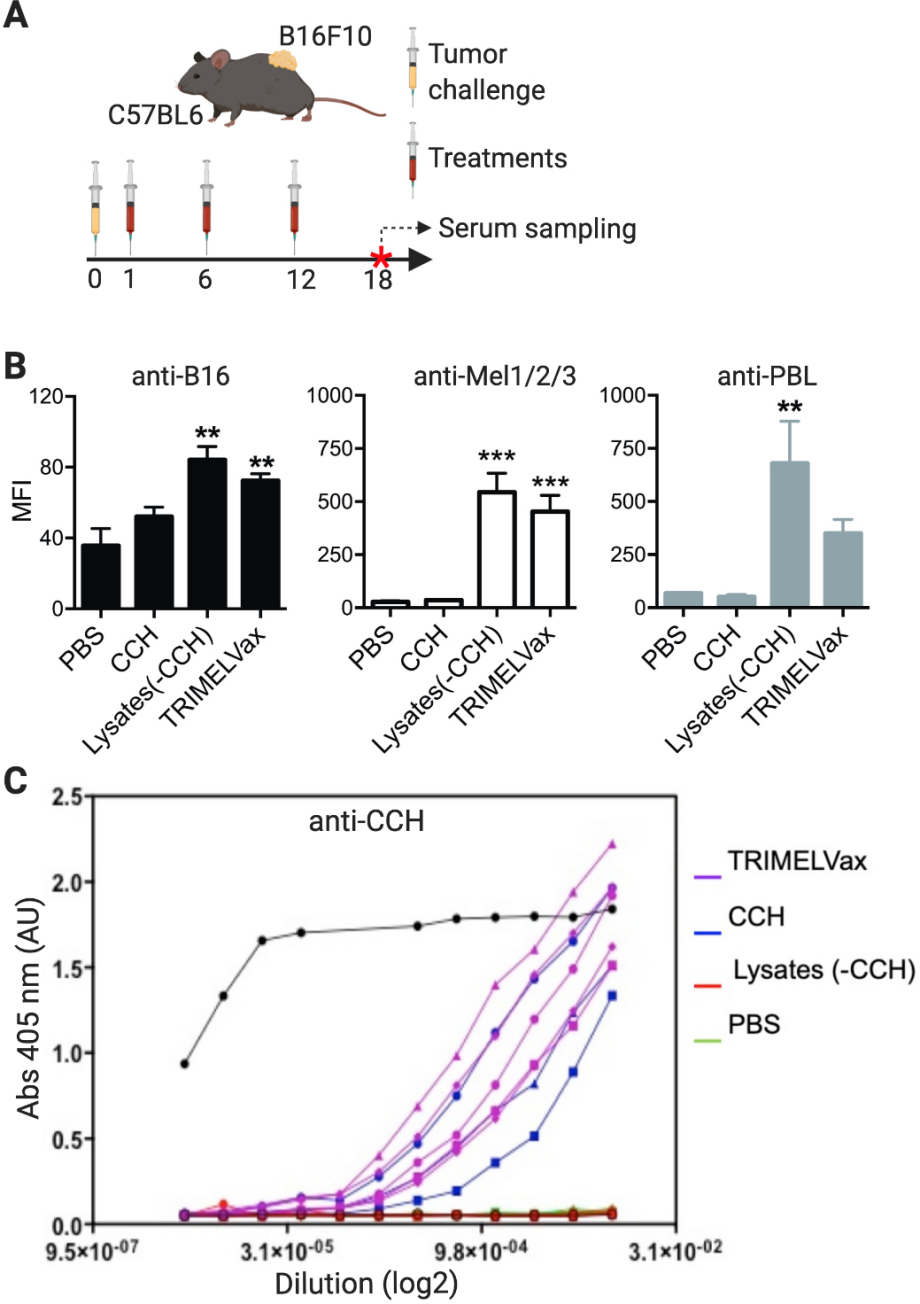
TRIMELVax induces humoral antimelanoma immune responses in vivo. (A) C57BL6 mice (three per group) were challenged subcutaneously with B16F10 cells and then subcutaneously treated as shown. Serum samples were collected 6 days after the last immunization and the presence of IgGs against B16F10, Mel1/Mel2/Mel3 cells, human peripheralblood leucocyte (PBL) (B) or *Concholepas concholepas* hemocyanin (CCH) (C) were tested by flow cytometry (B) or ELISA (C). (C) The curves represent the anti-CCH antibodies present in different dilution of serum obtained from animals treated with TRIMELVax (purple), CCH alone (blue), lysates without CCH (red) or vehicle/phosphate-buffered saline (PBS) (green). The black line corresponds to the detection of purified CCH (ELISA positive control). **p<0.01; ***p<0.001. MFI, Mean of fluorescence. Abs, absorbance.

TRIMELVax includes CCH, which is a strong adjuvant that induces both T-cell and humoral responses in different vaccine models.^26 30^ Therefore, we investigated the potential presence of antitumor antibodies in sera of vaccinated mice. Our results showed that sera from tumor-bearing mice vaccinated with either TRIMELVax or HS-melanoma cell lysates, but not CCH-treated mice, contains IgGs that bind B16F10 and Mel1/Mel2/Mel3 cells (figure 3A, B). Of note, we also observed a strong xeno-response induced by TRIMELVax, given that sera of vaccinated mice also reacts against human PBL (figure 3B). As expected, treatments with CCH alone or TRIMELVax, but not cell lysates alone, induced the generation of anti-CCH IgGs (figure 3C). These results suggest that TRIMELVax induce a strong humoral immune response against vaccine components, including MAAs.

### B16F10 tumor control by TRIMELVax requires HS pretreatment of melanoma cells and presence of CD4^+^ and CD8^+^ T cells

**Figure 4:**
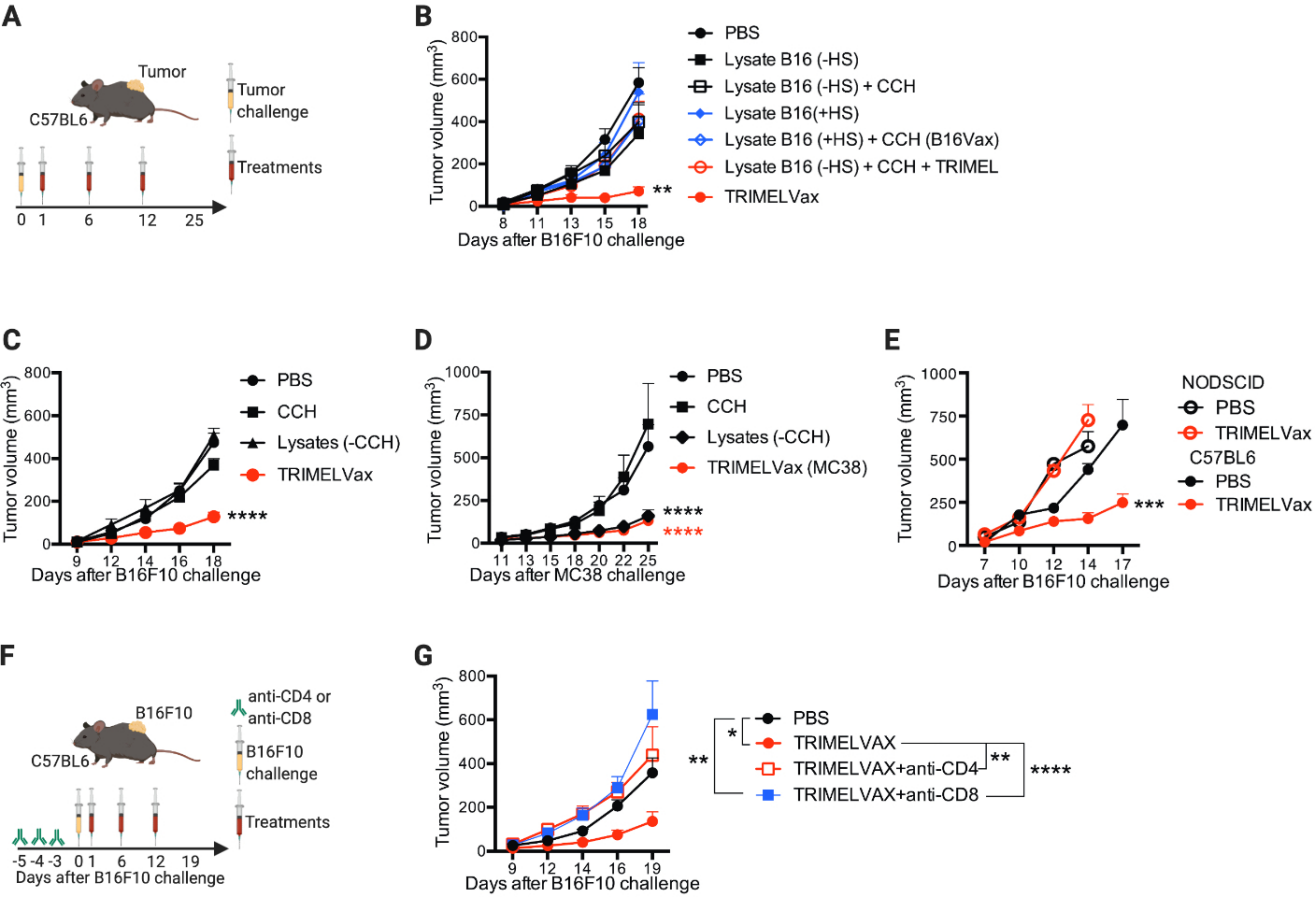
B16F10 tumor control by TRIMELVax requires heat shock (HS) pretreatment of melanoma cells and presence of CD4^+^ and CD8^+^ T cells. (A) Schematic representation of the therapeutic protocols used in (B–D). (B, C) Tumor growth curves of treated mice challenge with B16F10. Each point represents the mean tumor volume±SEM per group (n=7 (B) or=12 (C)). (D) Tumor growth curves of treated mice challenge with MC38. Each point represents the mean tumor volume±SEM per group (n=7). (E) Tumor growth curves of C57BL6 or NOD-SCID mice prophylactically treated with TRIMELVax or phosphate-buffered saline (PBS) and challenged with B16F10 cells. Each point represents the mean tumor volume±SEM per group (n=5). (F) Schematic representation of therapeutic protocols for CD4^+^ or CD8^+^ cell depletions and treatments used in (G). (G) Tumor growth curves of mice depleted or not for CD4^+^ or CD8^+^ cells challenged with B16F10 cells and treated with TRIMELVax. Statistical analysis was performed with two-way analysis of variance after Bonferroni correction. *p<0.05; **p<0.01; ***p<0.001; ****p<0.0001. CCH, *Concholepas concholepas* hemocyanin.

We tested antitumor effects of TRIMELVax in therapeutic vaccination schemes (figure 4A). It was observed that despite DAMP induction in HS-treated B16F10 cells, vaccination with HS-conditioned B16F10 cell lysates was not able to protect against B16F10 tumor growth, even in combination with CCH (B16Vax) (figure 4B). In contrast, TRIMELVax treatment efficiently controlled B16F10 tumor growth (figure 4B). Notably, the effect observed by TRIMELVax was abrogated if untreated B16F10-derived lysate was used as antigen source (4B). As observed in the prophylactic approach, the use of CCH was required for the antitumor effects mediated by TRIMELVax in the B16F10 melanoma model (figure 4C), but it was dispensable for the control of a more immunogenic tumor as the colon cancer MC38 model (figure 4D). As expected, it was observed that full immune competence was required for TRIMELVax efficacy, given that it did not impact tumor growth in NOD-SCID mice (figure 4E). Moreover, depletion of CD4^+^ or, in a major grade, of CD8^+^ cells abrogated TRIMELVax antitumor effect (figure 4F, G), suggesting that the vaccine may promote both CD8^+^ and CD4^+^ T-cell-mediated immunity.

Our results indicated that all components of TRIMELVax were required for an efficient activation of cellular and humoral responses and for controlling tumor growth in a CD8^+^ and CD4^+^ T-cell-dependent manner.

### TRIMELVax inhibits tumor growth and increases survival of B16F10 tumor-bearing mice even in the absence of anti-PD-1 therapy

**Figure 5:**
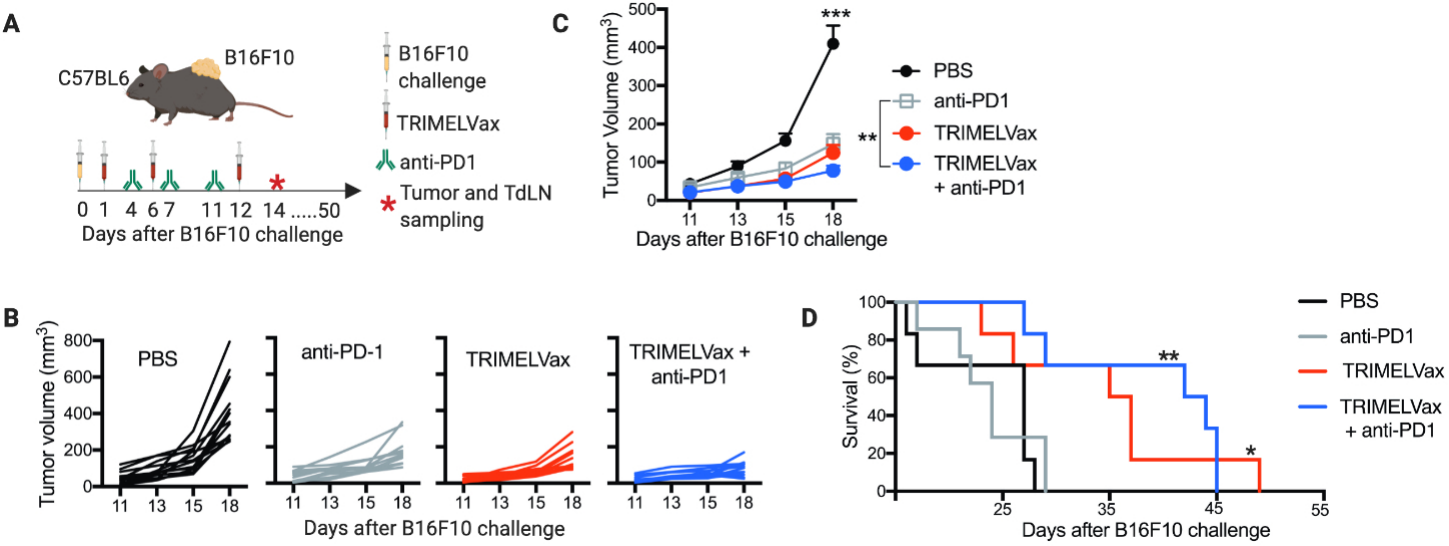
TRIMELVax controls tumor growth and increases survival of B16F10 tumor-bearing mice even in the absence of anti-PD-1 (programmed cell death protein 1) therapy. (A) Schematic representation of the therapeutic protocols for TRIMELVax/anti-PD-1 combinatory treatments. On day 14 after tumor challenging (asterisk), tumors and tumordraining lymph nodes (TdLNs) were sampled for 4–5 mice per group for further experiments. (B) Tumor growth curves of individual mice. (C) Each point represents the mean tumor volume±SEM per group (n=14). Statistical analysis was performed with two-way analysis of variance after Bonferroni correction. **p<0.01; ***p<0.001. (D) Kaplan-Meier curves for mice survival analysis (n=6–7 per group). The median survival time (in days after tumor challenge) per group is: phosphate-buffered saline (PBS), 27; anti-PD-1, 24; TRIMELVax, 36; TRIMELVax+anti-PD-1, 43. Statistical analysis was performed with log-rank (Mantel-Cox) test. *p<0.05; **p<0.01; ***p<0.001.

As anti-PD-1 monotherapy have shown partial protective effects in B16F10 tumors,^16^ we investigated whether the combination of TRIMELVax with anti-PD-1 could improve their immunotherapeutic potentials. Thus, we challenged C57BL6 mice with B16F10 cells and then treated with TRIMELVax, anti-PD-1 monoclonal antibodies or its combination (figure 5A). We observed that both single treatments independently inhibited initial tumor growth with similar efficacy, while the combinatory regimen induced a slightly better inhibition of tumor growth than anti-PD-1 monotherapy (figure 5B, C). However, despite the initial tumor growth control, the survival of anti-PD-1-treated animals was similar to the mock-treated ones (figure 5). Remarkably, TRIMELVax treatments alone or in combination with anti-PD-1, induced a significant improvement in the survival of tumor-bearing mice (figure 5D).

### TRIMELVax promotes tumor infiltration of cDC1 and PD-1^lo^ CD8^+^ T cells

**Figure 6:**
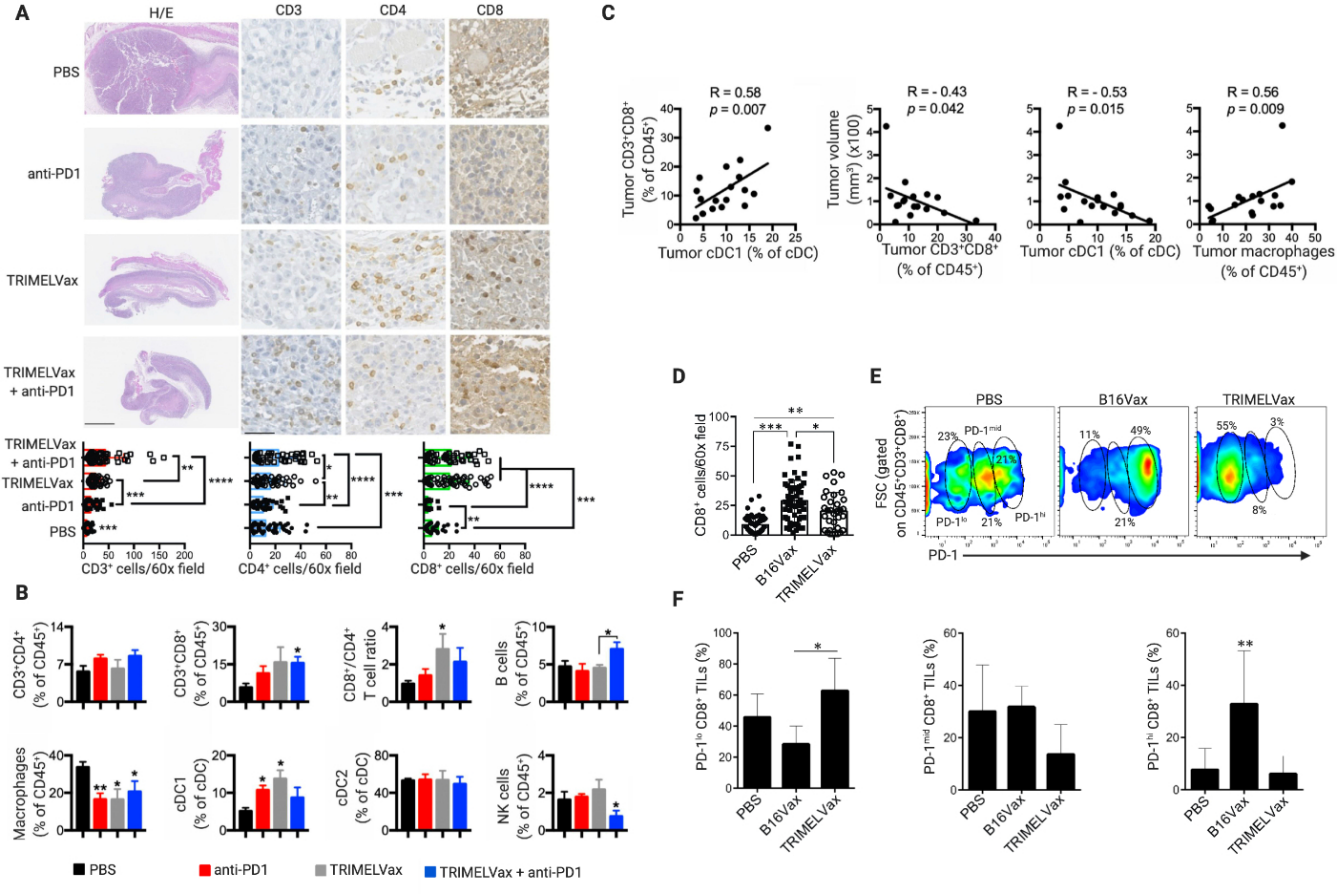
TRIMELVax promotes tumor infiltration of conventional type 1 dendritic cell (cDC1) and programmed cell death protein 1 (PD-1)^lo^ CD8^+^ T cells. (A) Immunohistochemistry analysis for CD3^+^, CD4^+^ and CD8^+^ tumor infiltrating cells of animals treated as described in figure 5. H&E representative photomicrographs of full tumors (left panels; scale bar: 1 mm), and for CD3^+^, CD4^+^ and CD8^+^ immune staining of selected tumor areas (scale bar: 40 µM). Dot plot/bar graphs show the quantification of immune cell infiltration by 60× field of tumors. (B) Tumors were sampled from mice treated as described in figure 5 and immune cell frequencies (among CD45^+^ cells) were analyzed by flow cytometry. Bar graphs represent average±SEM per group (n=4–5). (C) Percentage of intratumor CD8^+^ T cells as a function of the percentage of intratumor cDC1 (left); and tumor volume as a function of the percentage of different immune cells in tumors. Pearson’s r correlation values and p values are shown. (D) Dot plot/bar graphs show the quantification of CD8^+^ cells by 60× field of tumors of animals therapeutically treated with TRIMELVax, B16Vax or phosphate-buffered saline (PBS). (E, F) Flow cytometry analysis of PD-1 expression on CD8^+^ tumor-infiltrating lymphocytes (TILs) of animals treated as in (D). The percentage of PD-1^lo^, PD-1^mid^ and PD-1^hi^ CD8^+^ TILs is showed (n=4–6 animals/group). Statistical analysis was performed with two-way analysis of variance after Bonferroni correction. *p<0.05; **p<0.01; ***p<0.001; ****p<0.0001.

Associated with its better antitumor activity, TRIMELVax induced a higher tumor infiltration of CD3^+^, CD4^+^ and CD8^+^ cells than anti-PD-1 monotherapy, while TRIMELVax/anti-PD-1 combination generated higher tumor infiltration of CD4^+^ cells than each treatment alone (figure 6A). Flow cytometry analysis of tumors and TdLNs from vaccinated mice, sampled 14 days after tumor challenge, was performed to evaluate whether immune cell compartments were differentially affected by treatments. At that timepoint, both TRIMELVax and anti-PD-1 treatments induced increased frequency of intratumor cDC1s but not cDC2s (figure 6B). Also, we observed a decrease of intratumor macrophages frequency in all treated groups as compared with the PBS-control group (figure 6B). Unlike anti-PD-1 treatment, TRIMELVax induced potent tumor infiltration of CD8^+^ T cells, being CD8^+^/CD4^+^ T-cell ratio significantly enhanced by TRIMELVax (figure 6B). TRIMELVax/anti-PD-1 combination but not TRIMELVax alone showed a significant increase in the percentage of intratumor B cells (figure 6B). Additionally, we observed significant positive correlations between cDC1 and CD8^+^ T-cell intratumor frequency, and between tumor macrophage frequency and tumor size. On the other hand, significant negative correlations between intratumor CD8^+^ T-cell or cDC1 frequency and tumor size were also observed (figure 6C). The TdLN immune cell profile of treated mice is shown in online supplementary figure 6.

Finally, we wanted to investigate the CD8^+^ T-cell lineage phenotypes at tumor microenvironment, comparing their frequencies in tumor-infiltrating lymphocyte (TIL) from animals treated with TRIMELVax versus B16Vax. Although B16Vax therapeutic treatments lead to higher numbers of CD8^+^ TILs than TRIMELVax (figure 6D), this non-effective vaccine (figure 4B) lead to the accumulation of a higher proportion of PD-1^hi^ CD8^+^ T cells, whereas TRIMELVax promoted major proportion of PD-1^lo^ CD8^+^ T cells in tumors, a phenotype associated with prototypic effector cells required for tumor growth control (figure 6E, F).

## Discussion/conclusion

Clinical success of ICB therapies and their attractive combinatorial possibilities have generated enormous interest in cancer immunotherapy. However, limitations in objective responses have motivated a new impetus in the search for alternative/complementary immunological approaches, such as therapeutic cancer vaccines. Cancer vaccines are designed to trigger fresh-specific antitumor cytotoxic T lymphocyte responses, a prerequisite for clinical effectiveness of ICB.^16^

Despite the efforts, cancer vaccines can still be considered suboptimal. Such inefficient responses maybe related, at least in part, to improper delivery of TAA, induction of tolerance by dominant tumor peptides or immunological danger signal absence during immunization.^17–19^ Although auspicious, technical complexity of DC vaccine preparations has also limited their clinical implementation. However, having in mind the promising results of TRIMEL-loaded TAPCells in patients with melanoma,^8 9^ we intended to improve our approach using TRIMEL as a tumor cell lysate vaccine. Intact allogeneic whole-tumor cell and derived lysates have been extensively explored in different immunotherapeutic schedules against cancer.^6 8 18 19^ Although these vaccines allow for the presentation of multiple TAAs and showed promising results in animal models, they exhibited poor immunogenicity and some have shown very low efficacy in clinical trials.^18 19 31^ The inability of tumor cell lysates to stimulate a sustained immune response may be due in part to the presence of immune suppressive molecules within the lysate or the lack of appropriate neoantigens.^31^ Nevertheless, there is an increasing evidence that, at least under specific circumstances, stressing or damaging tumor cells can drive an inflammatory process that may culminate with the activation of a cell-mediated immune response.^32^ The stress-driven cell activation is now usually referred to as immunogenic cell death.^33^ Diverse treatment modalities, such as photodynamic therapy, high hydrostatic pressure, thermal shock, radiotherapy and some chemotherapeutic agents or oncolytic viruses, have been used to increase tumor cell immunogenicity. However, their proinflammatory effects and ability for induction of DAMPs vary and are dependent of multiple factors like tumor cell types and experimental settings.^32–34^

In this study, we demonstrate that TRIMELVax, a new vaccine based on HS-conditioned tumor cell lysates, inhibits tumor growth and increases B16F10 tumor-bearing mice survival. We have previously demonstrated the capacity of the HS-conditioned lysate TRIMEL to induce a mature phenotype in ex vivo generated DCs that are able to trigger antitumor immunity in patients with advanced melanoma and other tumors.^8 9 26^ Treatment of tumor cells with HS prior to cell lysis induces a variety of DAMPs^9 10^ that represent a requirement for the antitumor effect of TRIMELVax and promote major histocompatibility complex class I antigen presentation through stabilization of antigenic peptides by the chaperones role of HS proteins.^35^

In our experimental settings, although the HS treatment also induces DAMPs in B16F10 cells, and lysates derived from them induced murine DC activation in vitro, these lysates fail to protect mice for B16F10 tumor growth, unless it is combined with TRIMEL and CCH. Previously, we showed that lysates derived from HS-conditioned single cell lines are not always capable of promoting complete DC maturation; this is because not all the required DAMPs are present in every cell line.^9 10^ In contrast, TRIMEL seems to generate the appropriate combination of DAMPs capable of inducing an effective DC maturation.^8 9^ CCH has potent immunostimulatory effects, whose adjuvant properties could be partially explained by its carbohydrate-based high complexity and stability,^24^ which activate APCs via the interaction with the C-type lectin and Toll-like receptors (TLRs) such as mannose receptor and TLR4, respectively,^25^ strengthening innate and adaptive immune responses in mammals, making them useful in cancer immunotherapy.^26 30^

Notably, treatment of B16F10 tumor-bearing mice with TRIMEL/CCH or combined cell lysates in the absence of CCH did not prevent tumor growth (figure 2B), indicating that every vaccine component is crucial for induction of protective responses in our model. At this respect, we speculate that CCH may generate an acute inflammatory milieu at the injection site, promoting innate immune cell recruitment required for potent responses against weakly immunogenic tumors,^36^ while TRIMEL may act as an efficient source of immunogenic HS-induced DAMPs that enhance MAA cross-presentation.^9^ Finally, B16F10 HS-cell lysates provide a broad source of syngeneic MAA that may be stabilized by chaperons induced by the HS, as previously described by others.^35^

TRIMELVax capacity for efficiently controlling the weakly immunogenic and aggressive B16F10 melanoma tumor growth, prolonging tumor-bearing mice survival is, to the best of our knowledge, uncommon for vaccines targeting natural TAA based on non-genetically manipulated B16F10 cells or cell lysates. For example, it has been shown that HS-stressed and irradiated B16F10 cells prophylactically injected in mice did not produce tumor growth retardation, unless animals were immunized with LPS-stimulated BM-DCs loaded with stressed B16F10 cells.^37^ Another study showed that mitomycin-inactivated B16F10 cells did not protect mice against tumor growth, requiring genetic modifications to express Glycosylphosphatidylinisotol (GPI)-anchored interleukin 21 and secreting GM-CSF for success.^38^ Additional models of genetically modified B16F10-based vaccines, engineered to express GM-CSF,^17^ FLT3-L^14, 31^ or inducible T cell co-stimulator (ICOS) ligand,^15^ produce weak protective antitumor responses, unless they were combined with ICB.^39^

In contrast, monotherapy with TRIMELVax was sufficient to induce potent antitumor responses, even in the absence of anti-PD-1 antibodies. In fact, TRIMELVax/anti-PD-1 combination generated only marginal enhancing of TRIMELVax efficiency, although long-term effects in tumor-bearing animals cannot be discarded. In our model, treatment of tumor-bearing mice with anti-PD-1 alone induced tumor growth retardation, but did not have a significant impact on the overall survival, in line with other observations,^40 41^ which can be explained by acquired resistance to anti-PD-1/PDL1 therapies described in both mouse models and clinical trials.^3 42 43^

Furthermore, it has been shown that recruitment of CD8^+^ T cells into tumors requires the presence of intratumor cDC1 cells.^44^ Accordingly, TRIMELVax immunization resulted in a significant positive correlation between frequencies of intratumor cDC1 and CD8^+^ T cells. The observed inverse correlation between infiltration of these cell subtypes and tumor size reinforces the importance of inducing “hot tumors” for generating relevant antitumor responses.^5^ Additionally, our in vitro experiments showed that HS-treated cell lysates were preferentially phagocyted by cDC1 cells, which can efficiently cross-present MAA to CD8^+^ T cells in vivo.

On the other hand, we found a significant direct correlation between frequencies of tumor-associated macrophages and tumor size in vaccinated animals. In fact, increased tumor-associated macrophages infiltration in most solid tumors has been correlated with poor patient prognosis^45^ and associated with CD8^+^ T-cell tumor microenvironment exclusion and poor response to immunotherapy.^46^

The detection of antimelanoma antibodies in vaccinated mice may indicate that TRIMELVax complex composition appears to facilitate the exposition of surface and intracellular proteins in an acute inflammatory context, which may favor the triggering of humoral responses. Contribution of B cells to tumor immune response is for far less well investigated than T-cell-mediated responses.^47^ Nevertheless, humoral immune responses in patients with cancer have been found against a wide variety of cellular and extracellular proteins derived from transcription factors, cell cycle regulators, cell surface receptors or extracellular matrix.^47^

Chronic antigen stimulation occurring in persistent infections and cancer results in CD8^+^ T-cell misfunction. Exhausted CD8^+^ T cells showed decreased effector function and proliferative capacity, partly caused by the overexpression of inhibitory receptors such as PD-1. Recently, various CD8^+^ TIL populations have been described based on PD-1 expression levels: negative or low (PD-1^N^ or PD-1^lo^); intermediate (PD-1^mid^) and high (PD-1^hi^).^48 49^ PD-1^hi^ CD8^+^ T cells are characterized by an exhausted phenotype and lower production of proinflammatory cytokines than PD-1^lo^ CD8^+^ T cells. Moreover, PD-1^hi^ TILs are associated with worse prognosis, whereas high levels of PD-1^lo^ TIL indicate better clinical outcome for patients with cancer.^49^ Our results showed that TRIMELVax but not controls generated a strong antitumor immune response, reflected by the number of tumor infiltrating CD8^+^ T cells and also by enhanced prototypic PD-1^lo^ CD8^+^ effector T-cell infiltration preventing dysfunctional PD-1^hi^ CD8^+^ T-cell accumulation at the tumor site. Although we still do not have an explanation for this observation, it seems that the quality of T-cell priming is reflected in the tumor microenvironment, affecting the effector capacity of the TIL. On the other hand, we cannot rule out that the antigen specificity induced by TRIMELVax is different from that induced by B16Vax, affecting the quality of the resulting CTL.

Evidence shown in this report clearly demonstrates the therapeutic value of TRIMELVax, reflected in the immune-mediated tumor growth inhibition of the aggressive melanoma B16F10. Based on previous observations using TRIMEL-loaded DC vaccines, TRIMELVax translation to clinical trials will be favored by demonstrated TRIMEL properties as optimal supplier of a variety of shared MAAs, and source of necessary DAMPs to induce an appropriate in vivo antigen cross-presentation. Finally, the experimental model used in the present study provides an excellent system to further investigate cellular and molecular factors that may be essential to trigger protective tumor immune responses by active immunotherapy.

10.1136/jitc-2020-000999.supp2 Supplementary data
