# Supplementary material for: A heat-shocked melanoma cell lysate vaccine enhances tumor infiltration by prototypic effector T cells inhibiting tumor growth
Source: J Immunother Cancer. 2020 Jul 20;8(2):e000999. doi: 10.1136/jitc-2020-000999 (PMC7373330; doi:10.1136/jitc-2020-000999)
Supplement: Supplementary data [file jitc-2020-000999supp001.pdf]

Supplementary Material

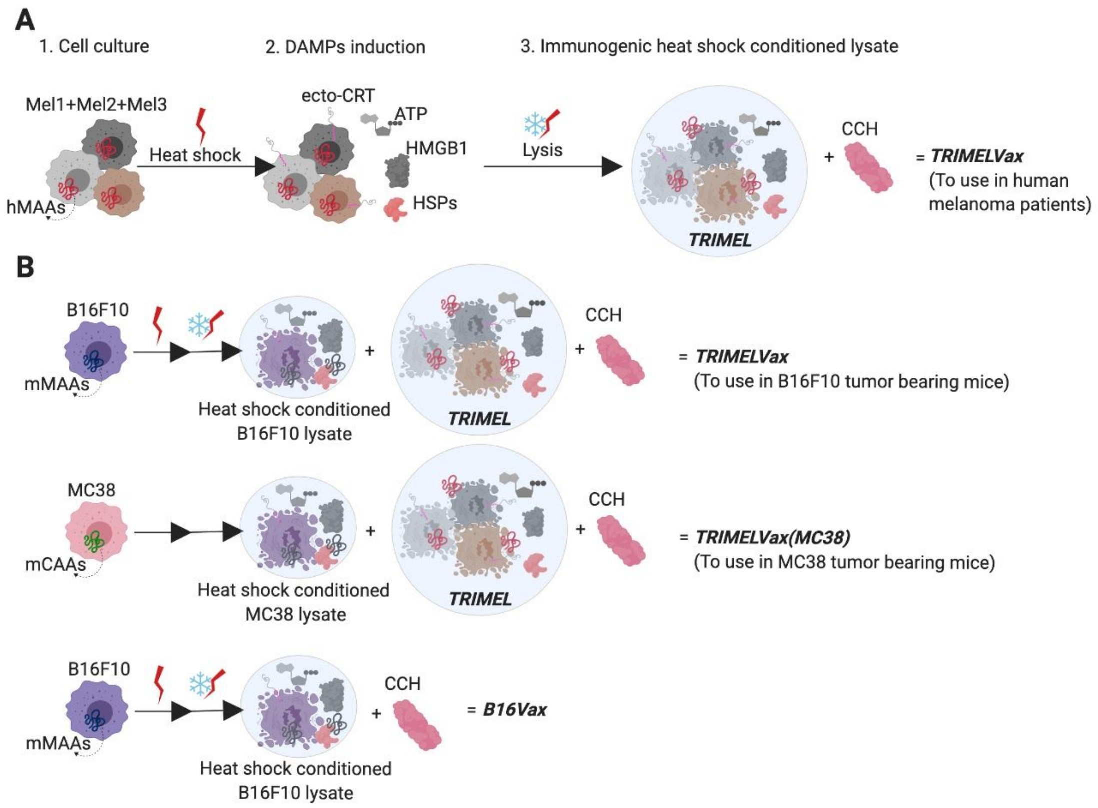

Supplementary Figure 1. TRIMELVax, TRIMELVax(MC38) and B16Vax vaccine compositions. (A) Key steps for the manufacture of the TRIMELVax vaccine. (1) Mel1, Mel2 and Mel3 human melanoma lines, positive for a wide panel of human melanoma associated antigens (hMAA), are cultivated until reaching the required number in a maximum confluence of 90%. (2) The melanoma cells are then harvested and subjected to a step of heat shock (HS), which induces the generation of several danger associated molecular patterns (DAMPs), such as the plasma membrane translocation of calreticulin (CRT), the release of ATP, HMGB1 and heat shock proteins (HSP). (3) Melanoma cells subjected to HS are lysed by freeze/thaw cycles to produce the immunogenic lysateTRIMEL. The combination of TRIMEL with the adjuvant CCH (hemocyanin of the mollusk *Concholepas*

concholepas) is called TRIMELVax. (B) Schemes showing the composition of the experimental vaccines TRIMELVax to evaluate antitumor activity in murine models of melanoma (B16F10 tumors, upper), colon carcinoma (MC38 tumors, middle) and B16Vax (lower). The HS-conditioned cell lysates of B16F10 and MC38 cells contain DAMPs as well as murine melanoma associated antigens (mMAAs) and murine colon cancer associated antigens (mCAAs), respectively.

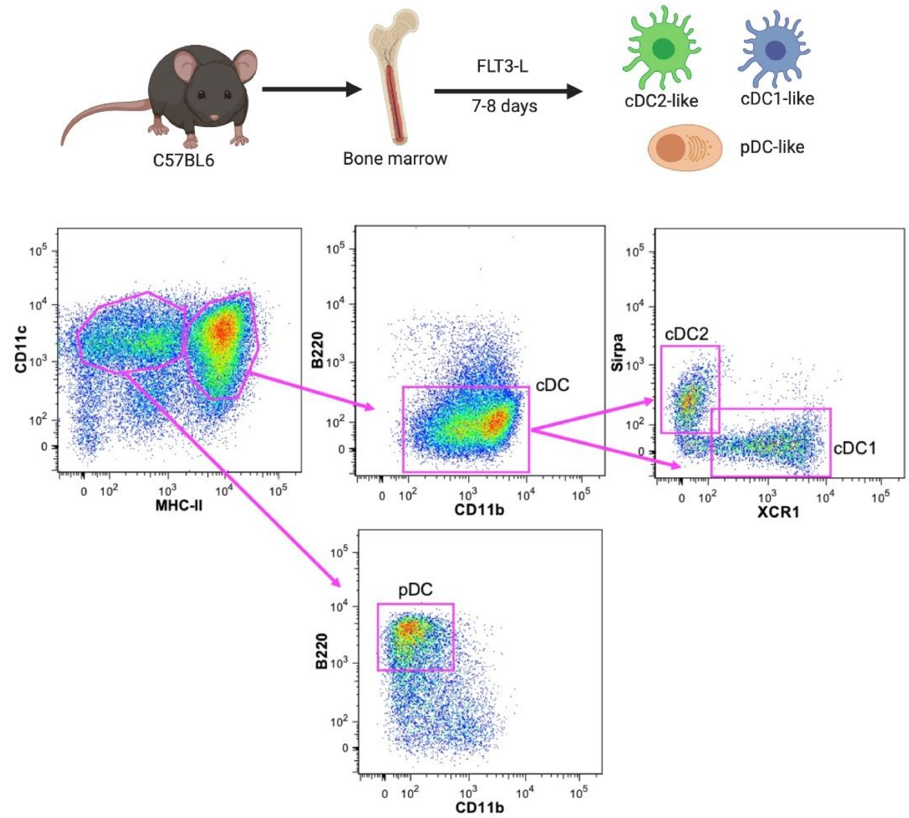

Supplementary Figure 2. Cell sorting strategy for *in vitro* differentiated cDC1, cDC2 and pDCs FL-DCs. Strategy of flow cytometry analysis of FL-DC generated *in vitro*. Gating was done on live cells and then on $CD11^{+}MHCII^{+}$ or $CD11^{+}MHCII^{low}$ cells.

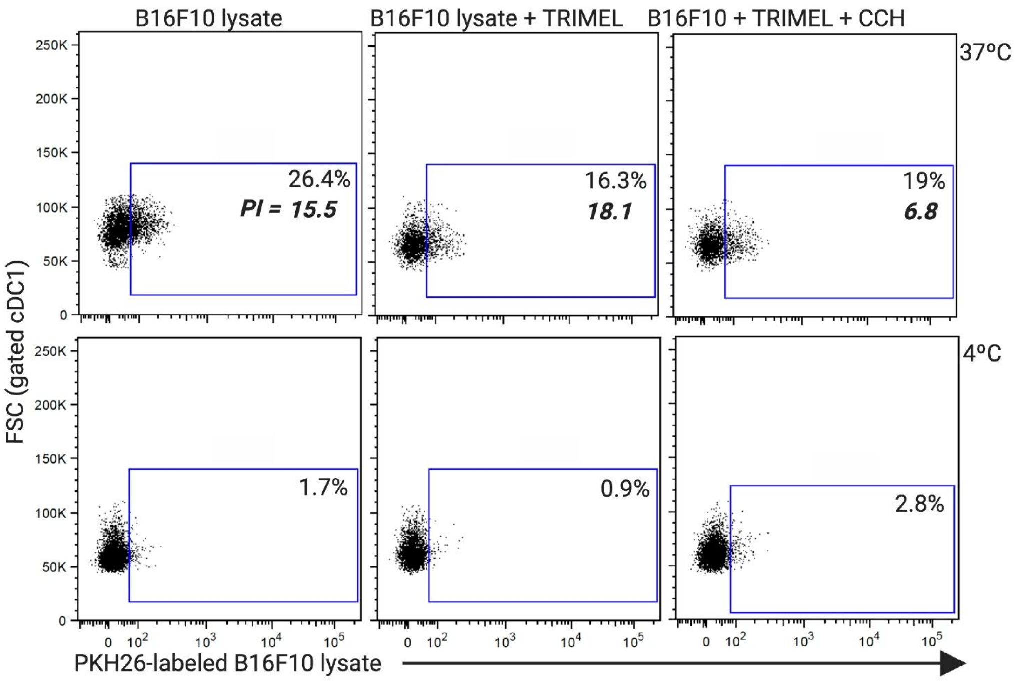

Supplementary Figure 3. TRIMELVax-stimulated cDC1 FL-DCs phagocyte heat shock conditioned B16F10 lysates. Representative flow cytometry dot plots show the uptake of PKH26-labeled B16F10 cell lysate or PKH26-labeled B16F10 cell lysate + TRIMEL, or PKH26-labeled B16F10 cell lysate + TRIMEL + CCH by cDC1 FL-DCs (ratio 1:1 at 37ºC for 3 hours). Gating was done on live cells and then on $CD11^{+}MHCII^{+}XCR1^{+}$ cells. As control cells were incubated at 4ºC.

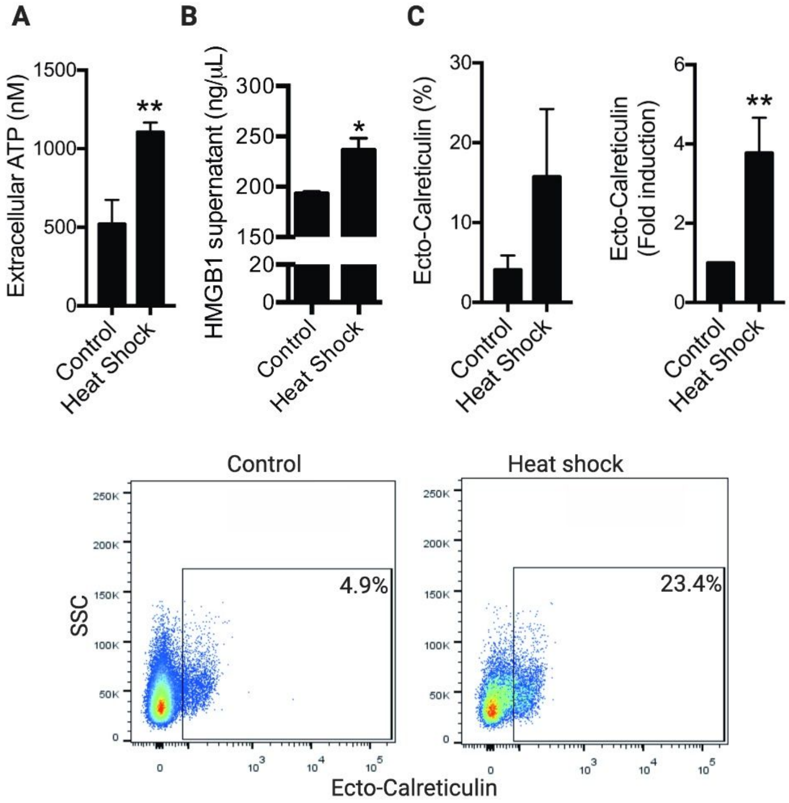

Supplementary Figure 4. Heat shock induces ATP and HMGB1 release and plasma membrane translocation of calreticulin in B16F10 cells. (A) ATP levels in supernatants of B16F10 cells treated with heat shock (HS) or not (control). (B) Culture supernatants were subjected to ELISA to measure HMGB1 expression levels. (C) FACS analysis of percentage of plasma membrane translocated calreticulin (ecto-CRT) in B16F10 cells treated or not with HS or not. Bars represent MFI averages increase in relation to control. Data represents at least 3 independent experiments. *$p < 0.05$; **$p < 0.01$.

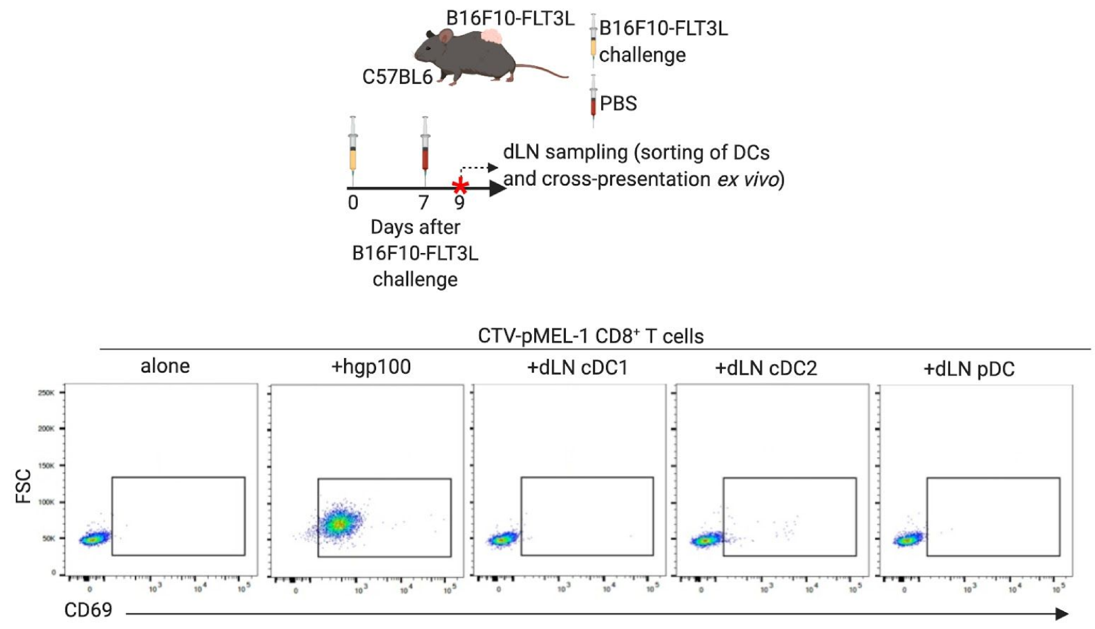

Supplementary Figure 5. Basal cross-presentation of the gp100 antigen of dLN-sorted DCs. On day 0, 2 × 10^6 B16F10-FLT3L cells were s.c. inoculated into C57BL6 mice. On day 7 after tumor challenging, mice were treated with PBS and after 2 days dLNs were sampled and used to get sorted cDC1, cDC2 and pDC cells. pMEL-1 $CD8^+$ T cells were co-cultured with dLN-sorted DCs, or stimulated with the hgp100 peptide (positive control) or kept unstimulated (negative control). The activation of the $CD8^+$ T cells was determined by CD69 expression after 3 days of coculture.

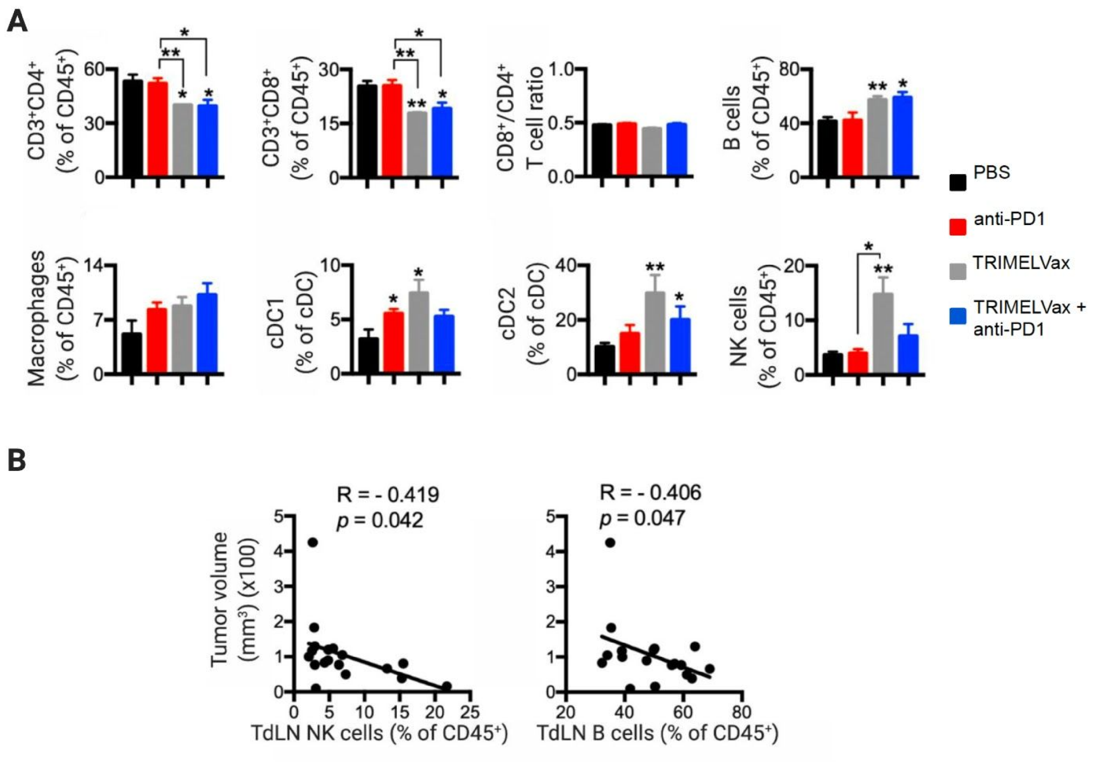

Supplementary Figure 6. TRIMELVax induces an increase in the frequency of NK cells and B cells in the TdLNs. (A) Tumor draining lymph nodes (TdLNs) were sampled from mice treated as described in Figure 5 and immune cell frequencies (among $CD45^+$ cells) were analyzed by multiparametric flow cytometry. Bar graphs represent average +/- SEM per group (n = 4-5). Statistical analysis was performed with two-way ANOVA after Bonferroni correction. * $p < 0.05$; ** $p < 0.01$ (significantly different from PBS control treatment, or as it indicated). (B) Tumor volume as a function of the percentage of TdLN NK cells and B cells. Pearson R correlation values and *p* values are shown.
