# Supplementary material for: A heat-shocked melanoma cell lysate vaccine enhances tumor infiltration by prototypic effector T cells inhibiting tumor growth
Source: J Immunother Cancer. 2020 Jul 20;8(2):e000999. doi: 10.1136/jitc-2020-000999 (PMC7373330; doi:10.1136/jitc-2020-000999)
Supplement: Supplementary data [file jitc-2020-000999supp002.pdf]

A heat shocked melanoma cell lysate vaccine enhances tumor infiltration by prototypic effector T cells inhibiting tumor growth

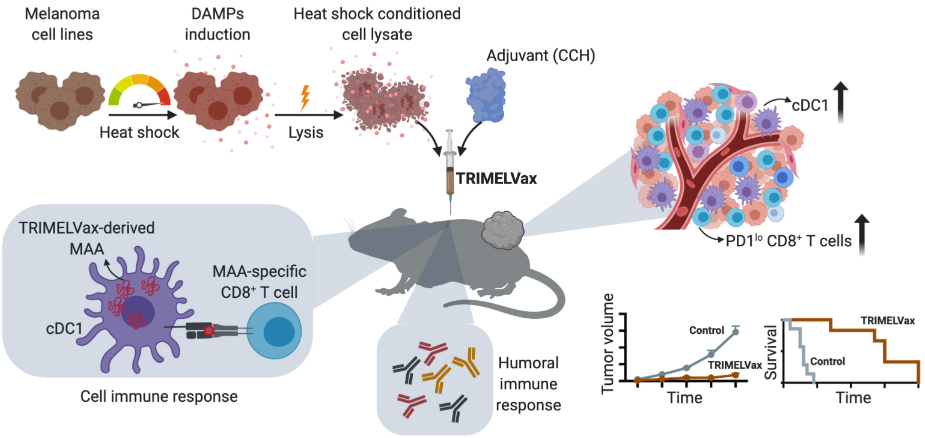

Authors

María Alejandra Gleisner, Cristián Pereda, Andrés Tittarelli, Mariela Navarrete, Camila Fuentes, Ignacio Ávalos, Fabián Tempio, Juan Pablo Araya, María Inés Becker, Fermín E. González, Mercedes N. López, Flavio Salazar-Onfray

Correspondence

fsalazar@u.uchile.cl

In Brief

The melanoma cell lysate based vaccine TRIMELVAx efficiently controls the weakly immunogenic and aggressive B16F10 melanoma tumor growth, prolonging tumor-bearing mice survival by enhancing tumor infiltration by cDC1 and prototypic PD-1^{lo} CD8^{+} effector T cells.
